# Reevaluation of the Pavlov Ratio in Patients with Cervical Myelopathy

**DOI:** 10.4055/cios.2009.1.1.6

**Published:** 2009-02-06

**Authors:** Kyung-Soo Suk, Ki-Tack Kim, Jung-Hee Lee, Sang-Hun Lee, Jin-Soo Kim, Jin-Young Kim

**Affiliations:** Department of Orthopedic Surgery, Kyung Hee University College of Medicine, Korea.; * Seoul Medical Center, Seoul, Korea.

**Keywords:** Cervical spine, Myelopathy, Pavlov ratio

## Abstract

**Background:**

This study was designed to reevaluate the effectiveness of the Pavlov ratio in patients with cervical myelopathy.

**Methods:**

We studied 107 patients who underwent open door laminoplasty for the treatment of cervical myelopathy between the C3 to C7 levels. We determined the Pavlov ratio on preoperative and postoperative cervical spine lateral radiographs, the vertebral body-to-canal ratio on sagittal reconstruction CT scans, and the vertebral body-to-cerebrospinal fluid (CSF) column ratio on T2-weighted sagittal MR images from C3 to C6. The severity of myelopathy was determined using the JOA score on both preoperative and postoperative images. The recovery rate was also calculated. The Pavlov ratio in plain radiographs from patients with myelopathy was compared with the ratio of the vertebral body to the spinal canal on CT and MRI.

**Results:**

The average Pavlov ratio between C3 and C6 ranged from 0.71 to 0.76. On CT scan, the average vertebral body-to-canal ratio between C3 and C6 ranged from 0.62 to 0.66. On MRI, the vertebral body-to-CSF column ratio between C3 and C6 ranged between 0.53 and 0.57. A positive correlation was noted between the Pavlov ratio and the vertebral body-to-canal ratio on sagittal-reconstruction CT (correlation coefficient = 0.497-0.627, *p* = 0.000) and between the Pavlov ratio and the vertebral body-to-CSF column ratio on MRI (correlation coefficient = 0.511-0.649, *p* = 0.000).

**Conclusions:**

We demonstrated a good correlation between the Pavlov ratio and both the vertebral body-to-canal ratio on CT and the vertebral body-to-CSF column ratio on MRI. Therefore, the Pavlov ratio can be relied upon to predict narrowing of the cervical spinal canal in the sagittal plane.

The developmental segmental sagittal diameter (the sagittal diameter from the posterior surface of the vertebral body to the nearest point of the corresponding spinal laminar line at the midvertebral level) is known to be unaffected by degenerative changes.^1^,2) Congenital narrowing of the spinal canal has proven to be a major risk factor for myelopathy in patients with cervical spondylosis.^3^-5) With regard to measurement of the diameter of the spinal canal, various tools can be employed, ranging from plain radiography (which allows for direct measurement) to digital equipment such as CT and MRI. Unfortunately, the latter two are not affordable or available to every patient. According to Herzog et al.1), the most accurate measurement of the developmental segmental sagittal diameter can be made on sagittal plane radiographs. Nevertheless, magnification error remains one of the major problems associated with plain radiography. Ratio measurements, especially the Pavlov ratio, are performed to overcome this obstacle. In the current study, we used the Pavlov ratio to evaluate cervical spinal stenosis in study subjects with cervical myelopathy. The purpose of this study was to reevaluate the effectiveness of the Pavlov ratio in patients with cervical myelopathy.

## METHODS

**Fig. 1:**
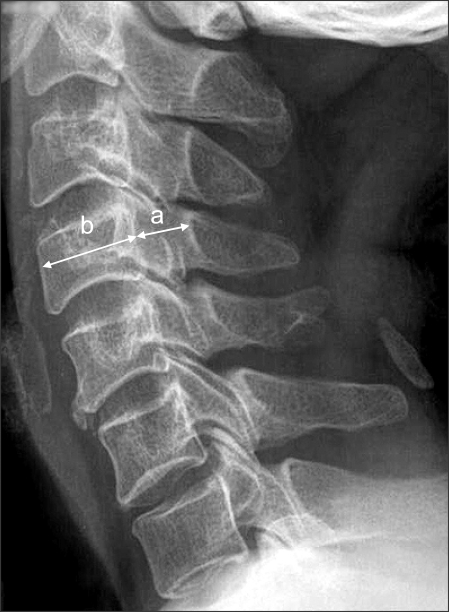
The sagittal diameter of the spinal canal (a) is measured from the posterior surface of the vertebral body to the nearest point of the corresponding spinal laminar line. The sagittal diameter of the vertebral body (b) is measured at the midpoint between the anterior surface and the posterior surface. The spinal canal/vertebral body ratio is determined using the formula a/b.

**Fig. 2:**
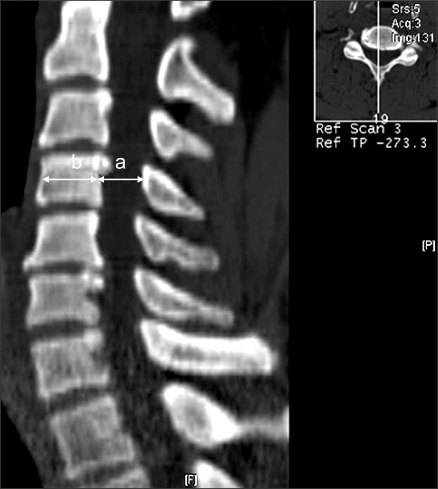
On the CT image, the diameters of the spinal canal (a) and vertebral body (b) are measured at the midvertebral level on sagittal-reconstructed CT images. The spinal canal/vertebral body ratio is determined using the formula a/b.

**Fig. 3:**
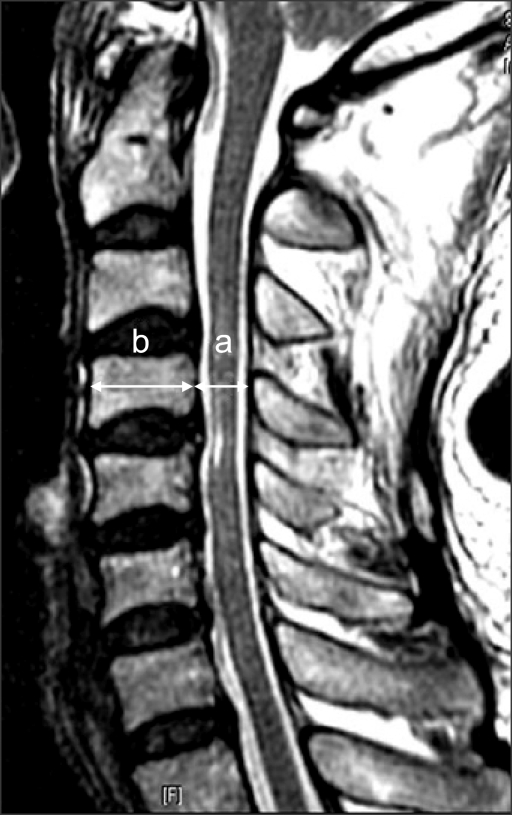
On the MR image, the sagittal diameters of the CSF column (a) and vertebral body (b) are measured at the midvertebral level on T2 sagittal images. The CSF column/vertebral body ratio is determined using the formula a/b.

The study population in this retrospective study consisted of 107 consecutive patients with cervical myelopathy who had undergone expansive open-door laminoplasty (Hirabayashi technique) for nerve compression between the C3 and C7 levels between August 2001 and August 2006. The mean patient age was 57.3 years (range, 35 to 80 years). There were 71 men and 36 women. Preoperative diagnoses included 77 cases of cervical myelopathy and 30 cases of posterior longitudinal ligament ossification. Preoperatively and postoperatively, the Pavlov ratio was measured at C3-C6 on lateral radiographs (Fig. 1). Preoperatively, sagittal-reconstruction CT scans were examined to calculate the vertebral body-to-canal ratio (Fig. 2). In order to measure the ratio on MRI, the vertebral body-to-cerebrospinal fluid (CSF) column ratio was calculated at C3-C6 based on T2-weighted sagittal MR images (Fig. 3). The recovery rate was calculated using preoperative and postoperative JOA scores and the Hirabayashi formula: [(postoperative JOA score-preoperative JOA score) × 100/(17-preoperative JOA score)]. The spinal canal expan-sion rate [(postoperative Pavlov ratio-preoperative Pavlov ratio) × 100/preoperative Pavlov ratio], devised by authors of this study, was also examined. We then investigated the correlation between the Pavlov ratio and the vertebral body-to-canal ratio measured from CT and MRI scans and between the JOA recovery rate and the spinal canal expansion rate. We performed Pearson correlation analysis using SPSS (SPSS for Windows Release 12.0, Chicago, Illinois) for the evaluation of the relationship between parameters. Statistical significance was defined as *p* < 0.05. The Pavlov ratio was measured with a digital measuring instrument on PACS. Three independent measurements were carried out by three orthopedic surgeons during a two-week interval. Pearson correlation analysis was done to examine the intra- and inter-observer reliability.

## RESULTS

**Fig. 4:**
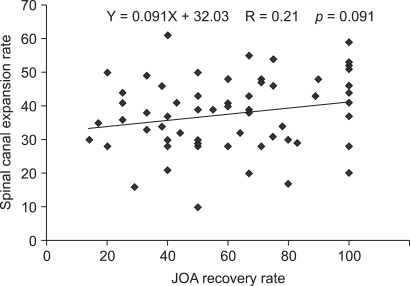
The correlation between JOA recovery rate and spinal canal expansion rate. The spinal canal expansion rate showed no significant correlation (r = 0.21, *p* > 0.05) with JOA recovery rate.

The mean Pavlov ratio for C3-C6 ranged between 0.71 and 0.76. The vertebral body-to-canal ratio on CT scans ranged between 0.62 and 0.66. The average vertebral body-to-CSF column ratio on MRI scans ranged between 0.53 and 0.57 (Table 1). The Pavlov ratios and values from CT were correlated with each other (correlation coefficient = 0.0497-0.0627, *p* = 0.000), as were the Pavlov ratios and values from MRI (correlation coefficient = 0.511-0.649, *p* = 0.000). A highly significant correlation was noted between the CT values and the MRI values (correlation coefficient = 0.707-0.816, *p* = 0.000). The average JOA score was 11.1 (range, 4 to 16) preoperatively and 15.0 (range, 8 to 17) at the last follow-up. Hence, the average recovery rate was 62.4%. The mean spinal canal expansion rate at C3-C6 was in the range of 31.7% to 50.5%. No meaningful correlation was noted between the JOA recovery rate and the spinal canal expansion rate (Fig. 4).

With regard to the intraobserver reliability, the mean Pearson correlation coefficient was 0.994 (range, 0.990 to 0.998), indicating few intraobserver errors. Interobserver errors were found to be minor, with a Pearson correlation coefficient of 0.990 (range, 0.980 to 0.996).

## DISCUSSION

CT and MRI are currently preferred for the assessment of cervical spinal canal stenosis, but historically the evaluation of cervical spinal stenosis has been based on plain lateral radiograph measurements of the diameter from the posterior surface of the vertebral body to the corresponding spinal laminar line. However, radiography is associated with magnification errors due to the distance from the patient to the film and the space from the film to the x-ray tube. Therefore, authors have suggested ratio analysis as a solution to magnification errors. Chrispin and Lee6) reported in their study of patients with myelopathy that the spinal canal area was smaller than the spinal body area and that cervical myelopathy was more likely to develop when the former was ≤ 85% of the latter. Ehni7) measured the sagittal diameter of the spinal canal and the anteroposterior diameter of the vertebral body on the assumption that the two measurements can be directly compared, as the spinal canal height and the vertebral body height are the same at a given level. Their results were as follows: in normal patients, the sagittal diameter of the spinal canal was similar to or greater than the anteroposterior diameter of the vertebral body; when the former was 80% of the latter, the probability of spondylolytic myelopathy was increased; when the former was 50% to 70% of the latter, spondylolytic myelopathy was almost inevitable. According to Pavlov et al.8), if the ratio of the sagittal distance of the spinal canal to the anteroposterior diameter of the vertebral body is ≤ 0.82, then cervical spinal stenosis is present. Magnification errors can be avoided using the Pavlov ratio, which also has the advantages of diagnostic efficiency and economic affordability compared with MRI and CT. However, the Pavlov ratio also carries some disadvantages. The sagittal diameter of the spinal canal was found to decrease with age in a previous radiographic evaluation study.4) In addition, considering that osteophyte formation generally occurs around the intervertebral disc, the values measured at the midvertebral level may not reflect the impact of the osteophyte in the diagnosis of spondylolytic myelopathy.9) Recently, Blackley et al.10) and Moskovich et al.11) reported that the Pavlov ratio was not necessarily associated with spinal stenosis due to the variability in the size of the vertebral body. Herzog et al.1) reported that the Pavlov ratio had a high sensitivity, but produced many false positive results. Meanwhile, Hukuda et al.12) reported that the Pavlov ratio was lower in patients with cervical myelopathy than in normal people. This claim was based on the observation that cervical myelopathy patients had not only narrower cervical canal diameter, but also wider vertebral body diameter than did ordinary people on sagittal plane radiographs. Chen et al.13) attributed cervical myelopathy to congenital cervical spinal stenosis based on their findings that Chinese men with cervical myelopathy had significantly lower Pavlov ratios, irrespective of age. In the current study of 107 patients with cervical myelopathy, the results obtained were congruent with those of Pavlov et al.8), with a ratio of 0.716 to 0.770.

Herzog et al.1) described a remarkably high correlation between the sagittal diameter of the cervical spinal canal derived from radiological images and that measured on CT images. In this study, we were able to correlate the Pavlov ratio with the vertebral body-to-canal ratio measured on sagittal reconstruction CT (correlation coefficient = 0.467-0.602, *p* = 0.000). A positive relation was also noted between the Pavlov ratio and the vertebral body-to-CSF column ratio derived from MR images (correlation coefficient = 0.602-0.620, *p* = 0.000). However, considering that the JOA recovery rate was not associated with the spinal canal expansion rate, we believe that various factors besides mechanical compression, such as poor blood flow and irreversible nerve cell injury, play a role in the development of myelopathy.^14^,15)

We noted a correlation between the Pavlov ratio mea-sured on lateral radiographs of the cervical spine and the vertebral body-to-canal ratio derived from sagittal reconstruction CT, as well as between the Pavlov ratio and the vertebral body-to-CSF column ratio measured on MR images. Therefore, we conclude that the Pavlov ratio is a useful tool in the assessment of cervical spinal stenosis.

## Figures and Tables

**Table 1:**
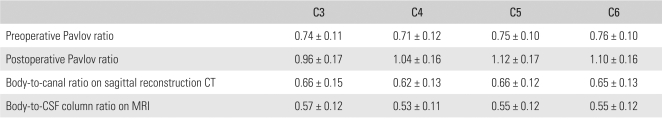
Mean Values for the Pavlov Ratio, Body-to-canal Ratio on Sagittal Reconstruction CT, and Body-to-CSF Column Ratio on MRI at Each Vertebral Level
